# ^177^Lu-PSMA-I&T Radioligand Therapy for Treating Metastatic Castration-Resistant Prostate Cancer: A Single-Centre Study in East Asians

**DOI:** 10.3389/fonc.2022.835956

**Published:** 2022-03-24

**Authors:** Ting Bu, Lulu Zhang, Fei Yu, Xiaochen Yao, Wenyu Wu, Pengjun Zhang, Liang Shi, Shiming Zang, Qingle Meng, Yudan Ni, Guoqiang Shao, Xuefeng Qiu, Shuyue Ai, Ruipeng Jia, Hongqian Guo, Feng Wang

**Affiliations:** ^1^ Department of Nuclear Medicine, Nanjing First Hospital, Nanjing Medical University, Nanjing, China; ^2^ Department of Urology, Nanjing Drum Hospital, Nanjing University, Nanjing, China; ^3^ Department of Urology, Nanjing First Hospital, Nanjing Medical University, Nanjing, China

**Keywords:** ^177^Lu, prostate cancer, metastatic castration-resistant prostate cancer (mCRPC), prostate-specific membrane antigen (PSMA), radioligand therapy (RLT)

## Abstract

**Purpose:**

There is increasing evidence for convincing efficacy and safety of ^177^Lu-labled prostate-specific membrane antigen (PSMA)-targeted radioligand therapy (PRLT) for metastatic castration-resistant prostate cancer (mCRPC). However, data are not available regarding the feasibility of ^177^Lu-labled PSMA-targeted RLT in East Asians. The present study summarized the first experience with ^177^Lu-PSMA-I&T therapy for mCRPC in China.

**Methods:**

Forty consecutive patients with mCRPC were enrolled from December 2019 to September 2021. Eligible patients received ^177^Lu-PSMA-I&T RLT at intervals of 8-12 weeks. Toxicity was assessed based on standardized physicians’ reports and the Common Toxicity Criteria for Adverse Events criteria. Response to PRLT was evaluated according to the changes of prostate specific antigen (PSA) response and imaging response. Quality of life (QOL), Karnofsky performance status (KPS) and pain (visual analogue scale, VAS) were also evaluated. The impacts of baseline parameters on the therapeutic effects were explored by univariate and multivariate logistic regression analyses.

**Results:**

All patients underwent a total of 86 cycles of ^177^Lu-PSMA-I&T (range: 1-5 cycles) with dosages of 3.70-14.43GBq per cycle, with a median of 8 months followed up. Six patients (15%) developed mild reversible xerostomia during follow-up, and 28 patients (70%) experienced grade 1-4 bone marrow dysfunction. Changes in PSA were assessed after therapy, accompanied by the partial response (PR) in 25 patients (62.5%), the stable disease (SD) in 5 patients (12.5%), and the progressive disease (PD) in 10 patients (25%), respectively. QOL, KPS (%) and VAS scores were improved significantly due to treatment (*P*<0.05). Overweight and elevated AST, ALP, and LDH were associated with poor outcomes.

**Conclusions:**

^177^Lu-PSMA-I&T achieves the favourable response and well tolerance in mCRPC, which associates with not only PSA decline but also with tumor remission including lymphadenopathy and bone metastasis. We also find that patients with overweight and high AST, ALP, and LDH should be cautious to undergo the PRLT. Large-cohort studies are warranted to confirm the initial findings and elucidate the survival benefit of the treatment.

## Introduction

According to cancer statistics, prostate cancer (PCa) was the third most common cancer in the United States in 2020 (1). In contrast, the incidence of prostate cancer in Asia is lower compared with western world, however, the incidence of high-risk PCa is likely to be increased, and the mortality of metastatic castration prostate cancer has been increased in the last decade. Lack of screening, limited treatment strategies in developing countries and subsequent high metastatic disease rates (30.5% in China and 23.7% in India) usually lead to reduced survival (2, 3). Once the disease has progressed to metastatic CRPC (also known as mCRPC), which is the leading cause of the morality, treatment options are limited (4, 5). Hence, novel strategies are needed to improve outcomes for these patients.

Prostate-specific membrane antigen (PSMA) is overexpressed in prostate cancer and is further increased in mCRPC. However, the biological characteristics of this type II transmembrane protein with glutamate-carboxypeptidase activity makes it an ideal target for radionuclide therapy (6–8). We previously showed that ^68^Ga-PSMA positron emission tomography/computed tomography (PET/CT) had higher sensitivity for detecting lymphadenopathy and visceral metastasis compared with multi-parametric magnetic resonance imaging, in terms of describing the clinical characteristics of intra-prostatic primary lesions including tumour size, shape, and location (9–12).

PSMA-targeted radioligand therapy (RLT) has been validated as a potential novel therapeutic strategy for mCRPC (13–16). A meta-analysis of 10 studies involving 455 mCRPC patients showed a pooled 68.0% decline of PSA after PSMA RLT (17). The latest VISION Clinical Trials showed that RLT with ^177^Lu-PSMA-617 prolonged PFS and OS when added to standard care in patients with PSMA-positive mCRPC (18). Previous studys showed that ^177^Lu-PSMA-I&T was produced conveniently and efficiently using an automated module, and had higher affinity towards prostate cancer cells and xenografts with higher PSMA expression (19). However, ^177^Lu-PSMA RLT has not been reported to treat Asian patients with mCRPC. We conducted a single-centre study to evaluate the treatment efficacy and safety of ^177^Lu-PSMA RLT in East Asian populations, and assessed the factors that may affect therapeutic efficacy. ^68^Ga-PSMA PET and ^177^Lu-PSMA-I&T SPECT/CT were also used to predict tumor response.

## Patients and Methods

### Study Population

**Figure 1:**
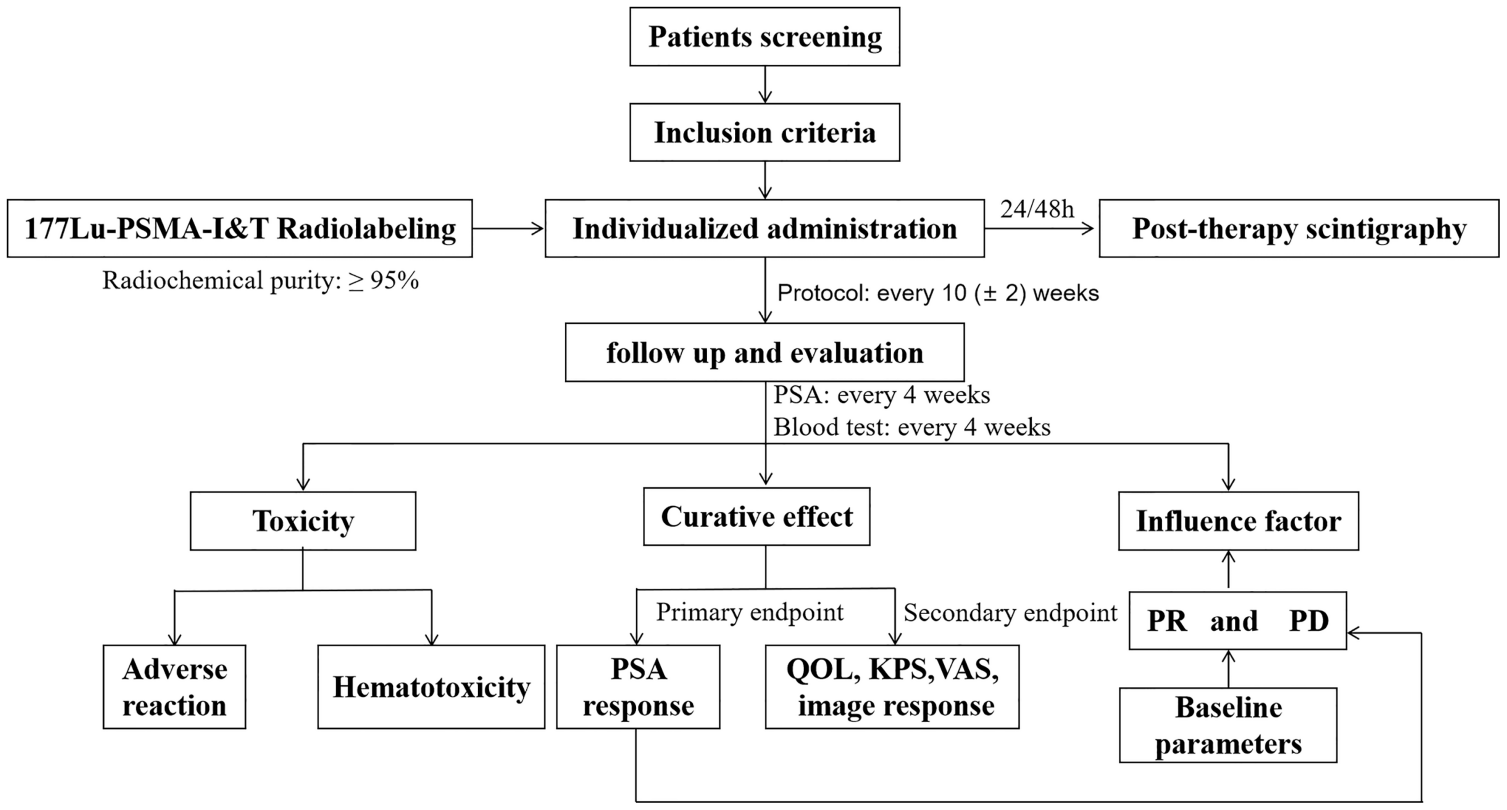
Flow chart of clinical trial.

A total of 40 consecutive patients (35 Chinese and 5 South Korean cases) with confirmed mCRPC were enrolled in this study from December 2019 to September 2021. All patients underwent ^68^Ga-PSMA-11 PET/CT in the outpatient clinic. At least three PSMA-avid lesions were detected by PET and the patients were transferred to the nuclear medicine ward. This prospective study was approved by the local ethics committee at Nanjing Medical University (KY20171208-03, Nanjing, China), and the trial was registered at clinicaltrials.gov (NCT04188587). All patients were fully informed of the precautions and possible adverse reactions and provided signed informed consent before treatment. The study flow chart is shown in **Figure 1**.

### ^68^Ga-PSMA-11 PET/CT Acquisition and Interpretation

PSMA-11 was purchased from ABX (Germany) and ^68^Ga-PSMA-11 was synthesised using an automation module (ITG, Munich, Germany). PET/CT (uMI780, United Imaging, China) was performed 45–60 min after injection of 111-185 MBq ^68^Ga-PSMA-11 (3-5 mCi). CT images were used for attenuation correction and accurate localization. PET imaging was acquired immediately after CT scan (matrix 256) with a 15.5 cm field of view and 3 min acquisition for each bed position.

Images were interpreted by a radiologist and a nuclear medicine physician who were blinded to the clinical characteristics and pathology. Tumour metastasis was evaluated by visual and semiquantitative analyses. Except for organs with physiologic uptake of PSMA (e.g., salivary gland, kidney, liver and small intestine), other sites with visual uptake greater than the average of the surrounding normal tissues (soft tissue/normal vertebral body) were regarded as PSMA-positive, suggesting local recurrence or metastasis. For all suspected pathological lesions (n=2329), the mean, maximum, and peak standardised uptake values (SUVmax, SUVmean and SUVpeak) of metastatic lesions and the tumour uptake volume (PSMA-TV/MTV) were calculated by VIOS with isocontours set at 45% of the maximum uptake within the respective focus. The SUVmax of liver parenchyma was also measured (mean value of 2 layers of normal liver parenchyma excluding lesions) to calculate the target/background ratio (T/B), and lesion PSMA uptake (TL-PSMA) was obtained by multiplying PSMA-TV by SUVmean. Whole-body PSMA TV (wbPSMA-TV) was the total volume of all PSMA-postive lesions, and whole-body TL-PSMA (wbTL-PSMA) was the summation of PSMA uptake by all lesions.

### ^177^Lu-PSMA-I&T and PRLT Protocol

High-purity lutetium chloride (^177^LuCl_3_) was obtained from ITG and PSMA-I&T was purchased from the Technical University of Munich (Germany) and was synthesized using an automation module (ITG). Only ^177^Lu-PSMA-I&T with more than 99% radiochemical purity was used in the clinical practice.

^177^Lu-PSMA-I&T was administered individually every 10 ( ± 2) weeks on average. Baseline haematological tests, liver and kidney function assays, dynamic salivary gland and renal imaging were performed, and the glomerular filtration rate was calculated prior to the initiation of treatment. Renal and salivary gland functions were therefore evaluated at baseline. ^177^Lu-PSMA-I&T was diluted to 20 ml with saline and infused intravenously within 20 min at a rate of 60 ml/h using a special infusion pump. To minimize treatment-related salivary gland damage, all patients received oral potassium perchlorate at 30 min and 4 and 12 h before intravenous ^177^Lu-PSMA-I&T to reduce salivary gland uptake. Patients also received vitamin C three times a day to protect salivary gland function, and folium sennae was used as a laxative to clear the intestines. In addition, patients continued standard treatment (hormone-deprivation treatment).

Whole-body scintigraphy and SPECT/CT were performed at 48 h post-injection of ^177^Lu-PSMA-I&T to evaluate tumour accumulation. SPECT images were displayed in three planes. A region of interest was drawn around the tumour and main organs and the ratio of tumour to liver was calculated to quantify tumour uptake. According to the regulations of the Chinese Office of Radiation Protection, all patients were discharged 48 h after treatment.

### Toxicity of PRLT

Toxicities were assessed following the Common Terminology Criteria for Adverse Events (CTCAE), version 5.0. Vital signs such as heart rate and blood pressure, and side effects such as nausea, vomiting, dyspnoea, and fatigue were observed for 4h during and after ^177^Lu-PSMA RLT. Blood, liver, and kidney function indexes were obtained at the fourth week after therapy. In addition to blood tests, side effects were observed during treatment and follow-up. Patients were asked about any symptoms related to RLT during their stay and at follow-up, including dry mouth, dry eyes, dysgeusia, weight loss, anorexia, fatigue, constipation, and dyspepsia.

### Treatment Efficacy Evaluation

All patients were observed to evaluate their quality of life (QOL), Karnofsky performance status (KPS, %) and pain (visual analogue scale, VAS) within 2-4 days after RLT. Serum PSA is the most important marker for evaluating the biochemical response to treatment. PSA levels were obtained before therapy and every 4 weeks subsequently. A decrease of ≥30% from baseline was considered as a partial response (PR), >25% increase in PSA above the baseline was defined as progressive disease (PD), and the change between PR and PD (< −30% and < +25%) was considered as stable disease (SD). ^68^Ga-PSMA-PET/CT at the 12th week after each therapy was also used to evaluate tumour response to ^177^Lu-PSMA. Soft tissue progression was defined according to RECIST 1.1 and bone disease progression was defined according to PCWG3 Consensus.

### Statistical Analysis

All statistical analyses were carried out and graphs were drawn using R (version 3.6.1) and GraphPad Prism software, respectively. Quantitative data with a normal distribution were expressed as mean ± standard deviation and data with a non-normal distribution were expressed as median (interquartile range, IQR). Wilcoxon’s signed-rank test was used to analyse treatment-related changes in safety and efficacy parameters. Univariate and multivariate logistic regression analyses were applied to identify the factors influencing the therapeutic effect. Statistical significance was established as *P* < 0.05.

## Results

### Patient Characteristics

Forty patients treated with ^177^Lu-PSMA-I&T were included in the study, baseline clinical characteristics are summarized in **Table 1**. All patients identified with mCRPC failed to standard treatment, who accompanied by multiple metastases (**Table 2**). Overall, 86 cycles were applied with a median of two cycles per patient (range 1-5), including two, three, four, and five cycles in sixteen, six, two, and two patients, respectively. Among the patients with only one cycle’ treatment, 10 patients gave up continuing treatment for various reasons, four patients with progressive diseases, five with financial difficulties, and one for the strict control of coronavirus. Individualized dose administration varied in all 40 patients, with the dosage per cycle ranging from 3.70-14.43GBq based on ECOG performance status, the extent of disease, and haematological, liver, and kidney function parameters. The median follow-up duration was 8 months (range: 2-20 months).

**Table 1:** Characteristics of patients’ cohort.

Parameters	Sub-group (n = 40)	Values
Age ^a^		67.8 ± 8.8 (range 45-87)
BMI ^a^		24.4 ± 2.9
Gleason score ^b^	6-7	2 (5)
	8-10	30 (75)
	Unkown	8 (20)
Karnofsky score, KPS ^b^	<80%	11 (27.5)
	≥80%	29 (72.5)
Eastern Cooperative Oncology Group, ECOG ^b^	0	15 (37.5)
	1	14 (35)
	2	11 (27.5)
Visual Analogue Scale/Score, VAS ^b^	No pain (<1)	5 (12.5)
	Mild (1-3)	19 (47.5)
	Moderate (4-6)	14 (35)
	Severe (7-10)	2 (5)
Prostate specific antigen, PSA (ng/mL) ^c^		76.60 (19.34,375.63)
Aspartate aminotransferase, AST (U/L) ^c^		21.00 (16.00,32.00)
Alanine aminotransferase, ALT (U/L) ^c^		14.00 (10.00,19.00)
Alkaline phosphatase, ALP (U/L) ^c^		119.00 (66.25,209.75)
lactate dehydrogenase, LDH (U/L) ^c^		261.00 (184.5,355.5)
Urea nitrogen, BUN (mmol/L) ^c^		5.59 (4.48,6.83)
Creatinine, CREA (μmol/L) ^c^		63.00 (52.50,71.50)
Anemia ^b^	Grade 1	8 (20)
	Grade 2	5 (12.5)
	Grade 3	2 (5)
	Grade 4	0
Leukopenia ^b^	Grade 1	8 (20)
	Grade 2	0
	Grade 3	0
	Grade 4	0
Thrombocytopenia ^b^	Grade 1	1 (2.5)
	Grade 2	0
	Grade 3	1 (2.5)
	Grade 4	0
SUVmax ^c^		36.37 (25.25,55.23)
SUVpeak ^c^		26.38 (17.97,38.68)
wbPSMA-TV ^c^		280.64 (77.19,725.97)
wbTL-PSMA ^c^		2460.10 (837.66,6269.84)
Prior systemic therapies ^b^	Prostatectomy	18 (45)
	Androgen-deprivation therapy	40 (100)
	Chemotherapy	19 (47.5)
	Abiraterone or enzalutamide or both	29 (72.5)
	Radiotherapy	14 (35)
	PARP inhibitor therapy	9 (22.5)

a: mean ± SD, b: n (%), c: median (interquartile range); BMI, body mass index; SUV, standard uptake value; PSMA, prostate specific membrane antigen; wbPSMA-TV, the whole-body tumor PSMA uptake volume; wbTL-PSMA, the PSMA uptake over all patient lesions in the whole-body.

**Table 2:** Extent of Disease on Baseline 68Ga-PSMA-11PET/CT.

Site of disease	No. Patients, n (%)	Supplement
Primary	16 (40)	
Lymph nodes	30 (75)	Cervical lymph node metastases in 7 cases (17.5%)
		Mediastinal and/or abdominal lymph node metastases in 29 cases (72.5%)
		Pelvic lymph node metastases in 5 cases (12.5%)
Bone metastases	36 (90)	<10, 5 (12.5%)
		10-40, 15 (37.5%)
		>40, 20 (50%)
Liver metastases	5 (12.5)	
Lung metastases	2 (5)	

### Post-Therapy Scintigraphy

**Figure 2:**
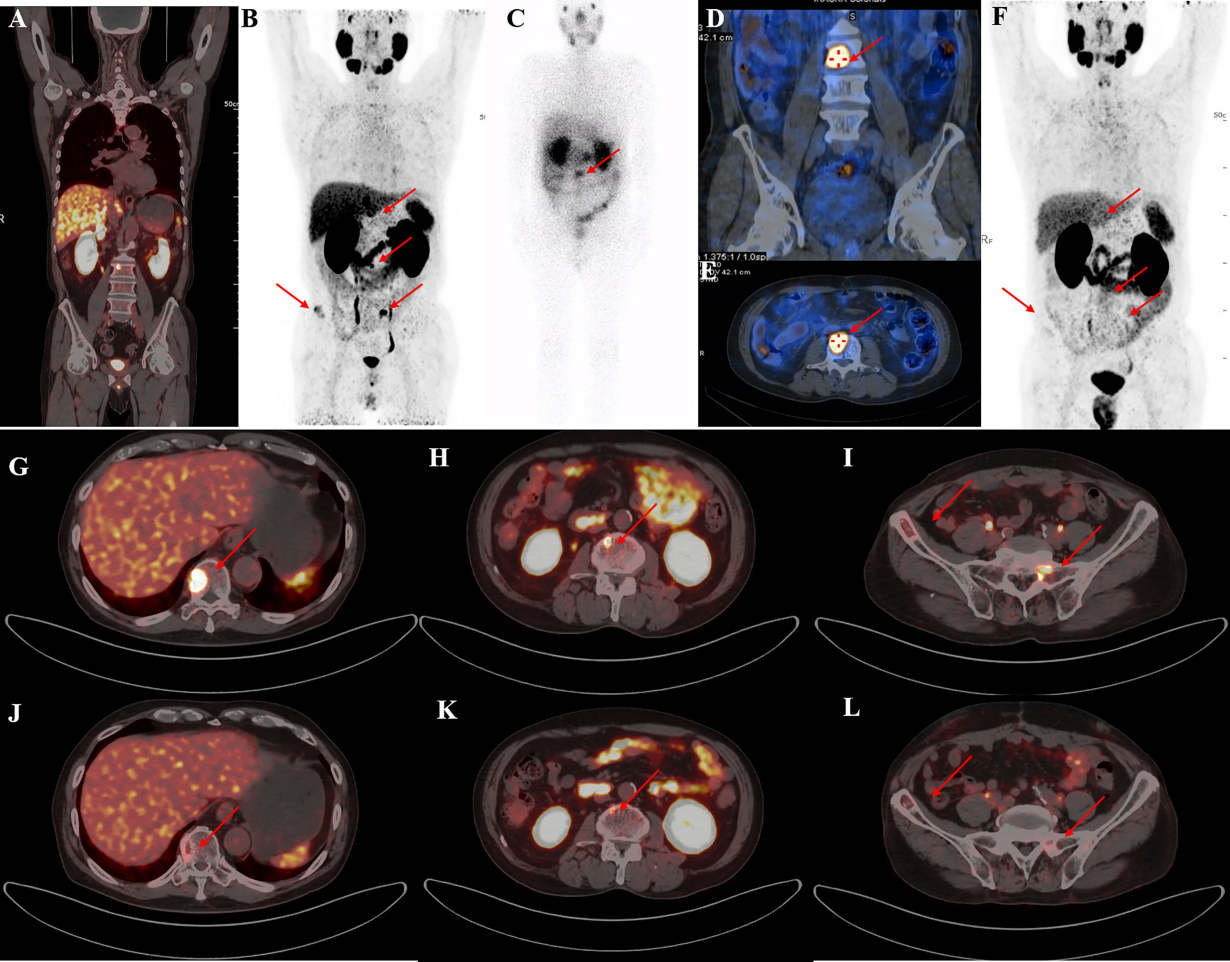
A representative 59-year-old patient with PSA decline ≥99% before and after Lu-PSMA-I&T therapy. **(A, B)** Baseline image and **(F)** image 3 months post-therapy. ^177^Lu-PSMA SPECT whole-body scan **(C)** showing higher contrast images 24 h post-injection with significant accumulation in the tenth thoracic vertebra sclerotic lesion. **(D, E)**: ^177^Lu SPECT image at 48 h showing destroyed and uneven density of L3 vertebrae with PSMA-avid lesion. Fusion image **(G-L)** showing reduced metastases in T10, L3, the right iliac, and sacrum compared with before therapy.

Post-therapy ^177^Lu-PSMA-I&T SPECT showed higher focal uptake in primary and metastatic lesions and lower uptake in normal liver and spleen. Adequate hydration and oral potassium perchlorate decreased the physiological uptake in normal lacrimal and salivary glands, small intestine, and kidneys. Radioactivity was mainly excreted rapidly *via* the kidneys, with uptake in the main organs significantly reduced or absent after 24h (**Figure 2**). PSMA-avid lesions were detected on ^177^Lu-PSMA-I&T images, with a median (IQR) tumour to liver ratio of 3.38 (2.50, 5.72) in the PR group compared with 2.80 (1.56, 4.70) in the PD group.

### Side Effects and Toxicity of PRLT

There were no transfusion-related complications or treatment-related deaths within 24 h after ^177^Lu-PSMA-I&T treatment, and no significant changes was seen in heart rate, blood pressure, or body temperature. Chest tightness with panic and shortness of breath occurred in one patient within 1-2 months after PSMA RLT treatment. Grade 1-2 fatigue and drowsiness were seen in 3(7.5%) and 2(5%) patients, respectively, on the second day after therapy. About 6 patients (15%) had mild to moderate xerostomia during follow-up.

Grade 1-4 impairment of bone marrow dysfunction occurred in up to 28 patients (70%). Before RLT, 5(12.5%) and 2(5%) patients had grade 2 and 3 anaemia, respectively, 8(20%) had grade 1 leukopenia, and 1(2.5%) had grade 1 and grade 3 thrombocytopenia. After treatment, 5 patients (12.5%) reported grade 3-4 anaemia, 1 patient (2.5%) reported grade 3 leukopenia, and 5 patients (12.5%) had grade 3–4 thrombocytopenia. In all 86 cycles, there was a significant decrease in haemoglobin [pre-therapy 110.00 (96.00, 125.00), post-therapy 107.00 (97.00, 119.00), *P*<0.05], erythrocyte counts [pre-therapy 3.56 (3.15, 3.87), post-therapy 3.33 (3.13, 3.79), *P*<0.05], leukocyte counts [pre-therapy 5.01 (4.00, 6.87), post-therapy 4.42 (3.80, 5.80), *P*<0.05] and platelet counts [pre-therapy 207.00 (158.20, 243.00), post-therapy 158.50 (120.50, 226.00), *P*<0.05].

No significant hepatotoxicity or nephrotoxicity was reported in the study. Aspartate aminotransferase (AST) and alanine aminotransferase (ALT) levels remained relatively stable after each cycle, while 2 patients diagnosed with liver injury with elevated AST, 1 patient developed transient grade 1 renal impairment with a slight increase in serum creatinine after therapy. All adverse events are listed in **Table 3**.

**Table 3:** Treatment related toxicity occurring up to 12 weeks after treatment cessation.

	Grade 1	Grade 2	Grade 3	Grade 4
	n (%)	n (%)	n (%)	n (%)
Fatigue	2 (5)	1 (2.5)	0 (0)	0 (0)
Lethargy	2 (5)	0 (0)	0 (0)	0 (0)
Nausea	0 (0)	0 (0)	0 (0)	0 (0)
Chest Distress	1 (2.5)	0 (0)	0 (0)	0 (0)
Xerostomia	3 (7.5)	2 (5)	1 (2.5)	0 (0)
Dry eyes	1 (2.5)	0 (0)	0 (0)	0 (0)
Bone Pain	19 (47.5)	3 (7.5)	2 (5)	0 (0)
Anemia	8 (20)	6 (15)	4 (10)	1 (2.5)
Leukopenia	8 (20)	2 (5)	1 (2.5)	0 (0)
Thrombocytopenia	2 (5)	2 (5)	4 (10)	1 (2.5)
Renal injury	2 (5)	0 (0)	0 (0)	0 (0)
Liver Injury	3 (7.5)	0 (0)	0 (0)	0 (0)

### Biochemical and Clinical Responses

**Figure 3:**
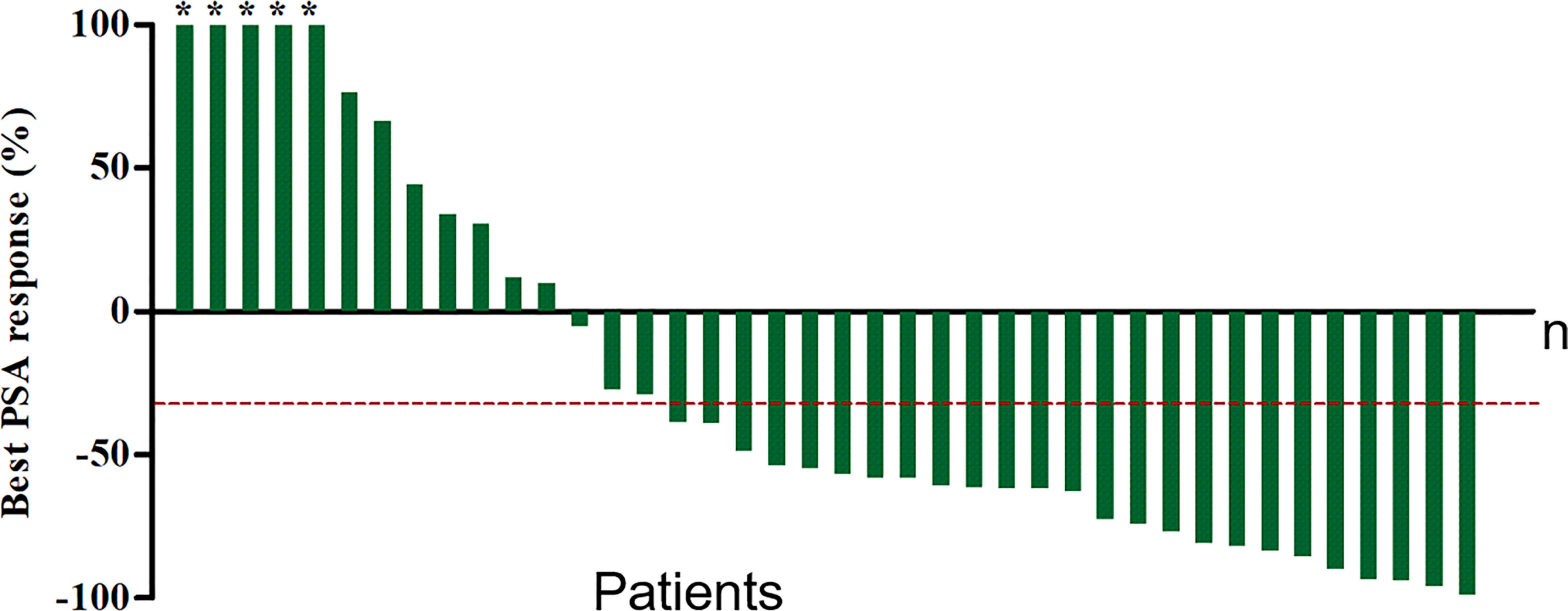
Waterfall plot of best PSA decline compared with baseline. Plots ordered from worst to best responder. Red dotted line represents 30% PSA decline. *The maximum PSA decline >90%.

At a median of 8 weeks after the first cycle of ^177^Lu-PSMA-I&T RLT, PSA was declined in 26 patients (65%) and PR was achieved in 18 patients (45%), indicating an improved efficacy for most patients. Furthermore, at the 8-week follow-up after the last cycle of therapy for all patients (including those who received only one cycle), the best PSA response showed that 28 patients (70%) had a PSA decline (*P*<0.05) (**Figure 3**). Overall, 10 patients (25%) had PD (>25% increase in serum PSA over baseline), 25 patients (62.5%) had PR (≥30% decrease in serum PSA). The number of patients achieving maximum PSA decline of >50% and >90% were 22, 4, respectively. At follow-up, 10 patients (25%) had developed clinical progression and 5 were dead (among them, 3 patients died of malignant tumor, the other 2 patients were because of heart and cerebral vascular disease).

`Of the 40 patients, 35 patients (87.5%) had pain at baseline and the VAS decreased compared with baseline after all cycles [median (IQR): baseline 3 (1, 4); after all cycles 1 (0, 2.25), *P*<0.05]. KPS, QOL were increased in 36 patients and were significantly improved after treatment [KPS: median (interquartile range): baseline 90 (77.5, 90); after all cycles 95 (80, 100), *P*<0.05; QOL: baseline 44 (40, 47.25); after all cycles 49 (46.75, 52.25), *P*<0.05].

### ^68^Ga-PSMA PET-CT for Evaluation of Tumour Response

**Figure 4:**
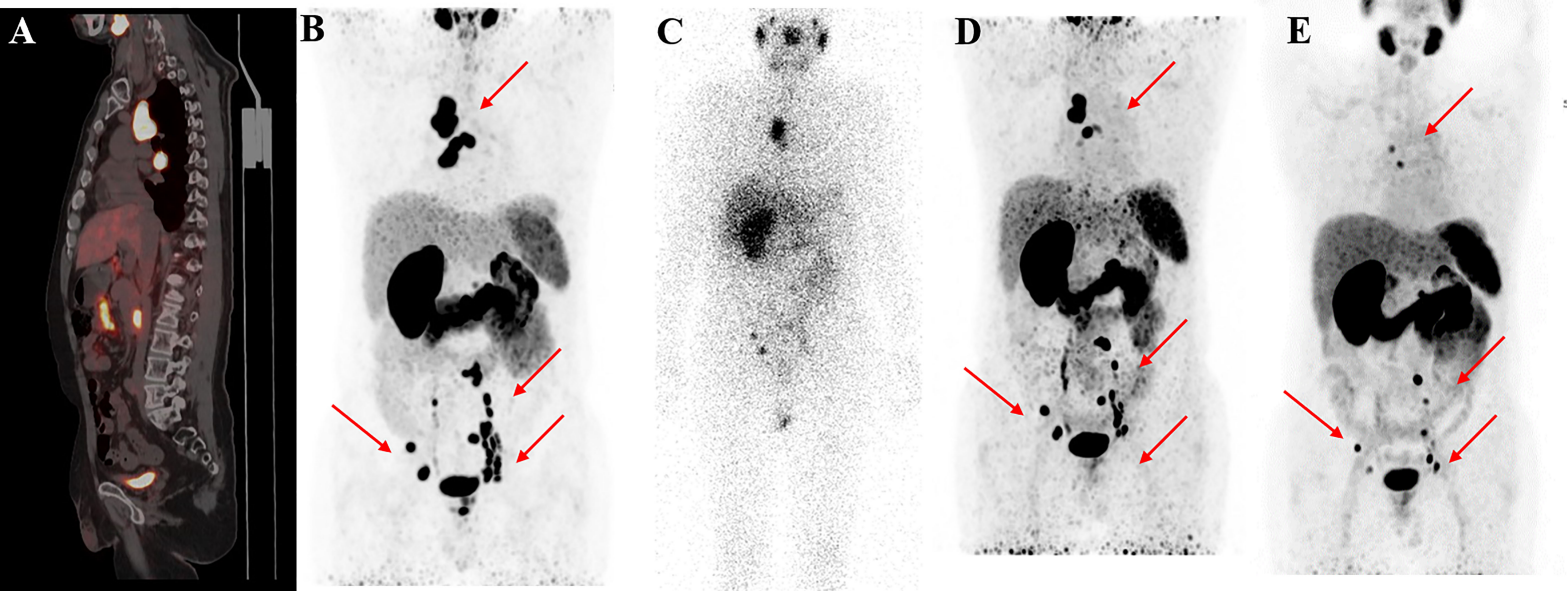
^68^Ga-PSMA-11 PET/CT in a 55-year-old patient who underwent four cycles of ^177^Lu-PSMA-I&T therapy. Baseline images **(A, B)** of multiple lymph node metastases in the right subclavian area, mediastinum, retroperitoneum, and pelvis. Post-therapy image **(C)** showing higher ^177^Lu-PSMA-I&T aggregation in primary and metastatic lesions. ^68^Ga-PSMA PET/CT after the first cycle **(D)** and after the third cycle **(E)** showing multiple decreased or disappeared metastases.

**Figure 5:**
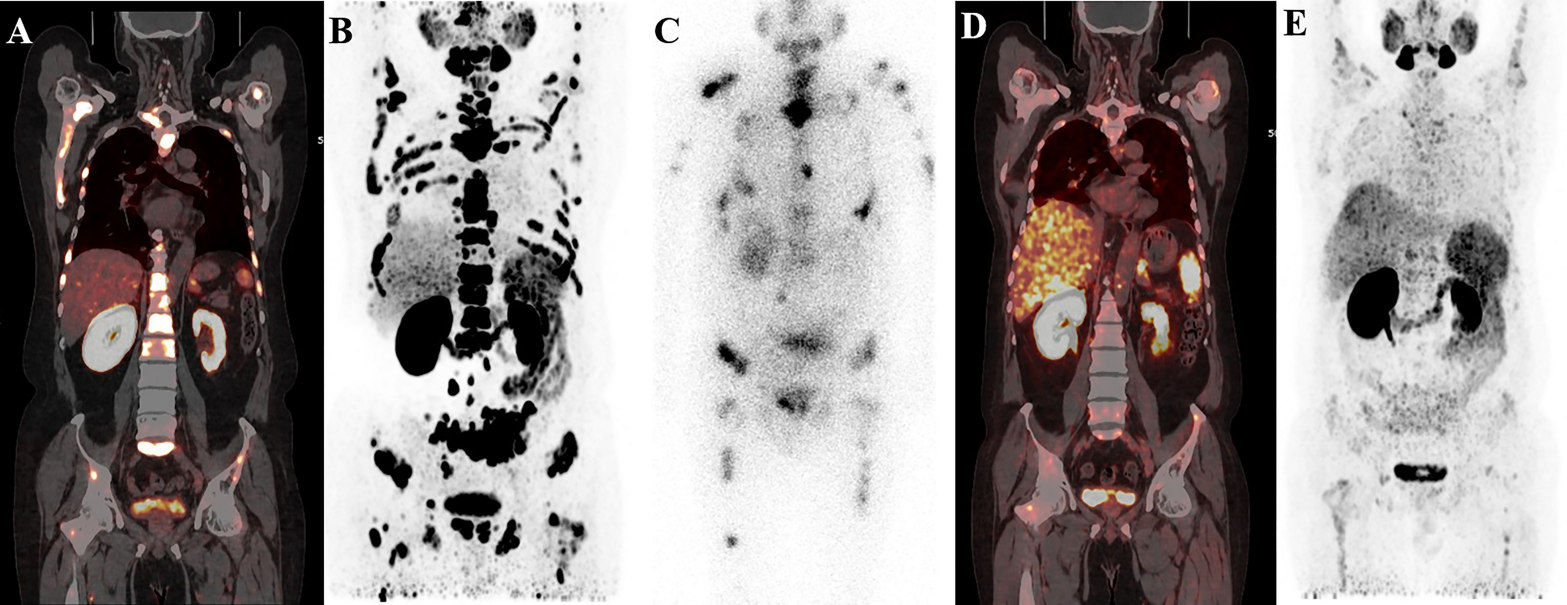
A 58-year-old patient with mCRPC with multiple systemic metastases. Baseline **(A, B)** ^68^Ga-PSMA-11 PET/CT image showing sclerotic bone metastases with higher PSMA expression. Post-therapy image **(C)** showing higher accumulation of ^177^Lu-PSMA-I&T in primary and metastatic lesions. Post-treatment image **(D, E)** showing extensive osteogenic bone metastases throughout the body, and reduced expression of PSMA compared with before therapy, but some weak remaining PSMA expression, suggesting that the treatment was effective.

**Figure 6:**
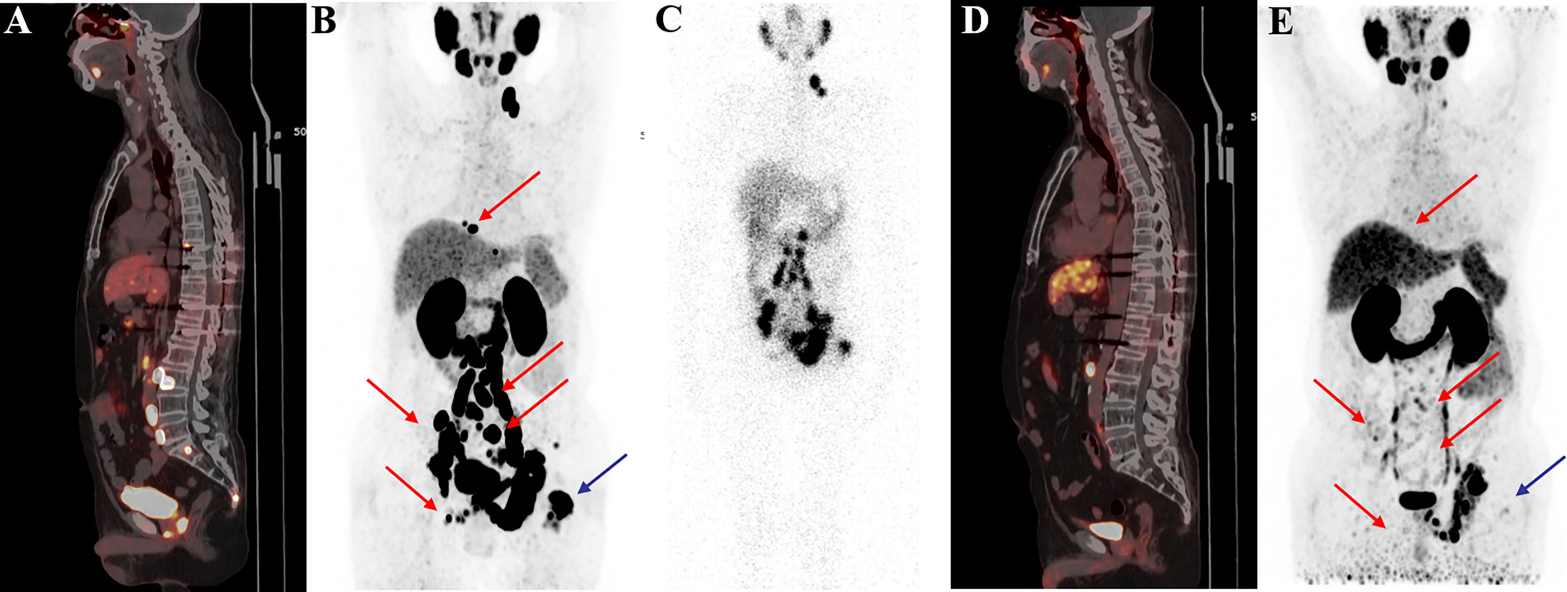
^68^Ga-PSMA-11 PET/CT in a 62-year-old patient with mCRPC. Baseline images **(A, B)** of multiple bone and lymph node metastases with increased PSMA expression. Post-therapy image **(C)** showing high ^177^Lu-PSMA-I&T aggregation in primary and metastatic lesions. Post-treatment image **(D, E)** showing multiple osteogenic metastases and reduced PSMA expression compared with before therapy. Metastatic foci in the left clavicle, abdominal aorta, and bilateral iliac paravascular lymph nodes were significantly reduced and PSMA expression was decreased, suggesting that the treatment was effective.

^68^Ga-PSMA PET-CT was performed in 23 patients followed by two or more cycles of ^177^Lu-PSMA-I&T. The median (IQR) SUVmax values of the target lesion before and after therapy were 27.7 (11.5, 44.7) and 20.27 (7.35, 35.92), respectively. ^68^Ga-PSMA PET revealed PD in 4 patients (17.4%). Eleven patients (47.8%) had PR with a better response for the significant decreased tumour size, reduced SUVmax, and disappearance of bone and lymph node metastases (**Figures 4**–**6**). One patient from Korea with multiple bone metastases achieved a ≥98% PSA decline after one cycle of ^177^Lu-PSMA-I&T and an almost complete response (**Figure 2**).

### Impacts of Baseline Parameters on Therapeutic Efficacy

Patients were classified into a remission and non-remission, and progression and non-progression groups according to PR and PD. Univariate logistic regression analysis of pretherapeutic signs [age, body mass index (BMI), KPS, serum PSA] and prior treatments confirmed that higher BMI was related to clinical progression [odds ratio (OR) and 95% confidence intervals (CI) 6.91 (1.43, 51.56)]. We also found that higher baseline serum AST, alkaline phosphatase (ALP), and lactate dehydrogenase (LDH) were associated with poorer outcomes (high levels of the above three biomarkers were negatively related to remission [OR (95%CI) 0.17 (0.03, 0.68) for AST, 0.20 (0.05, 0.79) for ALP, and 0.20 (0.05, 0.79) for LDH] and positively related to progression [11.77 (1.87, 231.47), 6.00 (1.24, 44.58), and 6.00 (1.24, 44.58), respectively)]. A higher BMI was also associated with clinical progression (OR (95%CI) 6.91 (1.43, 51.56) (**Table 4**). In addition, baseline imaging parameters including post-therapy SPECT/CT (T/B_SPECT/CT_), pre-therapeutic ^68^Ga-PSMA-11 PET-CT (e.g., PSMA-TV, TL-PSMA, SUVmax, SUVpeak, T/B_PET_, number of metastatic lesions, and presence of visceral metastasis) showed no significant correlation with response (**Table 5**).

**Table 4:** Univariate logistic regression analysis on basic factors associated with therapeutic effect.

Variants	Remission		Progression	
	OR (95% CI)	*P*	OR (95% CI)	*P*
Age				
<68	1		1	
≥68	1.45 (0.40,5.40)	0.57	0.51 (0.11,2.16)	0.37
Baseline BMI				
≤24	1		1	
>24	0.44 (0.12,1.61)	0.22	6.91 (1.43,51.56)	**2.75E-02**
Baseline KPS				
<90	1		1	
≥90	0.64 (0.16,2.36)	0.51	1.78 (0.41,9.54)	0.46
Prostatectomy				
Yes	1		1	
No	0.58 (0.16,2.12)	0.41	4.03 (0.92,21.84)	0.08
Abiraterone				
Yes	1		1	
No	0.89 (0.22,3.39)	0.86	0.75 (0.17,3.50)	0.70
Enzalutamide				
Yes	1		1	
No	0.50 (0.11,2.18)	0.35	0.69 (0.15,0.78)	0.67
Chemotherapy				
Yes	1		1	
No	1.13 (0.30,4.56)	0.87	1.33 (0.29,5.81)	0.71
Radiotherapy				
Yes	1		1	
No	0.90 (0.25,3.30)	0.86	1.31 (0.30,5.66)	0.70
Baseline PSA				
<76.60	1		1	
≥76.60	1.91 (0.53,7.31)	0.33	0.58 (0.13,2.46)	0.47
Baseline WBC				
<4.88	1		1	
≥4.88	1.91 (0.53,7.31)	0.33	0.58 (0.13,2.46)	0.47
Baseline RBC				
<3.6	1		1	
≥3.6	0.62 (0.16,2.23)	0.46	2.67 (0.61,14.26)	0.21
Baseline HGB				
<115.5	1		1	
≥115.5	0.95 (0.26,3.44)	0.94	1.50 (0.35,6.91)	0.58
Baseline PLT				
<211.5	1		1	
≥211.5	1.91 (0.53,7.31)	0.33	1.00 (0.23,4.30)	1.00
Baseline ALT				
<14.5	1		1	
≥14.5	0.33 (0.08,1.23)	0.11	3.05 (0.70,16.37)	0.15
Baseline AST				
<22	1		1	
≥22	0.17 (0.03,0.68)	**0.019**	11.77 (1.87,231.47)	**0.027**
Baseline BUN				
<5.59	1		1	
≥5.59	1.91 (0.53,7.31)	0.33	0.58 (0.13,2.46)	0.47
Baseline CREA				
<60	1		1	
≥60	0.95 (0.26,3.44)	0.94	0.88 (0.20,3.76)	0.86
Baseline ALP				
<126	1		1	
≥126	0.20 (0.05,0.79)	**0.0269**	6.00 (1.24,44.58)	**0.0403**
Baseline LDH				
<261	1		1	
≥261	0.20 (0.05,0.79)	**0.0269**	6.00 (1.24,44.58)	**0.0403**

WBC, leukocyte count; RBC, red blood cell count; HGB, hemoglobin; PLT, platelet count. Bold: Significance.

**Table 5:** Univariate logistic regression analysis on basic imaging factors associated with therapeutic effect.

Variants	Remission		Progression	
	OR (95% CI)	*P*	OR (95% CI)	*P*
SUVmax				
<36.37	1		1	
≥36.37	1.91 (0.53,7.31)	0.33	0.33 (0.06,1.43)	0.15
SUVpeak				
<26.38	1		1	
≥26.38	3 (0.81,12.24)	0.11	0.33 (0.06,1.43)	0.15
wbPSMA-TV				
<280.64	1		1	
≥280.64	1.24 (0.34,4.56)	0.74	0.58 (0.13,2.46)	0.47
wbTL-PSMA				
<2460.10	1		1	
≥2460.10	1.24 (0.34,4.56)	0.74	1 (0.23,4.30)	1.00
T/B_PET/CT_				
<8.06	1		1	
≥8.06	3 (0.81,12.24)	0.11	0.33 (0.06,1.43)	0.15
T/B_SPCET/CT_				
<3.20	1		1	
≥3.20	1.24 (0.34,4.56)	0.94	0.58 (0.13,2.46)	0.47
Number of lesions				
<40	1		1	
≥40	1.24 (0.34,4.56)	0.74	0.58 (0.13,2.46)	0.47
Visceral metastasis				
Yes	1		1	
No	0.76 (0.14,4.41)	0.75	2.79 (0.46,15.82)	0.24

T/B, the target/background ratio.

**Figure 7:**
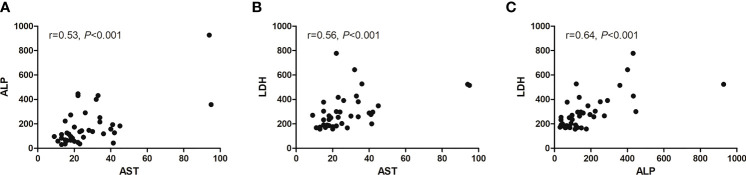
Scatter diagram showing correlation between three baseline serum biomarkers. **(A)** AST and ALP, **(B)** AST and LDH, and **(C)** ALP and LDH.

We assessed the relation among AST, ALP, and LDH with multivariate logistic regression analysis (**Figure 7**). Only AST and BMI were therefore included in the multivariate logistic regression analysis, and both were independent predictors of progression after RLT (**Table 6**).

**Table 6:** Multivariate logistic regression analysis on factors associated with disease progression after PRLT.

Variants	OR (95% CI)	*P*
Baseline AST		
<22	1	
≥22	11.68 (1.67,241.79)	**3.42E-02**
Baseline BMI		
≤24	1	
>24	6.85 (1.24,56.78)	**4.08E-02**

Bold: Significance.

## Discussion

The Vision Clinical trial showed that PRLT significantly improved treatment outcomes compared with standard medical treatment, and that ^177^Lu-PSMA-617 might be a new horizon for the treatment of mCRPC (20, 21). PSMA-I&T and PSMA-617 are the most widely studied PSMA inhibitory ligands in the field of prostate cancer treatment. There was no significant difference in curative effects and toxic reactions between the two radioactive ligands, but the average absorbed dose of ^177^Lu-PSMA-I&T is lower than that of ^177^Lu-PSMA-617 in lacrimal gland, which rarely causes symptoms of lacrimal gland dysfunction such as dry eyes and blurred vision (19). However, there are limited sporadic clinical studies on ^177^Lu-PSMA-I&T (19, 22). To date, PSMA ligand radionuclide therapy for prostate cancer has not been fully established for its widespread use in Asia. This study was to explore the clinical manifestations of ^177^Lu-PSMA-I&T in the East Asians.

The fixed activity of ^177^Lu-PSMA is usually used for PRLT, which has some merits for decrease the toxicity of the regimen, but might have some impact on treatment efficacy (15, 22). Herein, we speculated that higher activities per cycle are probably feasible and may increase the response rates and/or duration of response. Gaertner FC et al. reported that individual adaptations of therapy protocols based on diagnostic PSMA PET imaging before therapy might help to further promote efficacy and decrease the toxicity of RLT (23). For patients with large PSMA-positive tumor volumes, higher activities can be safely administered to maximize tumor biologically effective doses (BEDs) without exceeding the tolerable BED to the organs at risk, and for patients with severe tumor burden, the optimal activity can reach 14.84GBq (24). Thus, in this study, we conducted individualized administration based on pre-treatment PET and/or dosimetry studies.

In a large meta-analysis of 145 mCRPC patients who received PSMA RLT in Germany, 72% had PSA declines after the second cycle, which is similar to the current results (16). In this study, the therapeutic effects of ^177^Lu-PSMA-I&T on East Asian populations were systematically implemented. The results showed that 65% of patients had a PSA decline after the first cycle, and PSA continued to decrease during subsequent treatment. After the last cycle, 70% had a PSA decline. Probably because of differences in tumor staging among treated patients and differences in disease characteristics among ethnic groups, this considerable finding is slightly superior to results of another clinical experience with ^177^Lu-PSMA-I&T in Germany in PSA response. The percentage of patients achieving maximum PSA declines of 30%, 50%, and 90% were 47%, 38%, 11% in the previous study and 62.5%, 55%, 10% in this study, respectively (22).

^68^Ga-PSMA PET/CT is of great value for evaluating treatment response (14, 15, 25, 26). Based on baseline images and SUVmax of target lesions, four patients progressed with extensive metastases after two cycles, 8 patients had SD and 11 patients showed better response, with a significant reduction in tumour size and SUVmax. Furthermore, ^68^Ga-PSMA-PET/CT provides useful details about the target lesions, such as tumour size reduction and SUV decrease and the remain lesions. In short, ^177^Lu-PSMA-I&T RLT can be applied in patients with mCRPC, who fail to prior therapies.

In current study, significant physical and psychological improvements were observed after therapy. In addition to the changes in biochemical parameters and tumour size in images, VAS, KPS, and QOL were all enhanced after therapy. Notably, some patients with PD still showed pain relief and improved QOL, which was not consistent with a poor PSA response. We supposed that this could be due to a large tumour burden and visceral metastasis, and combined systematic treatment might further improve the outcome.

Treatment-emergent adverse events under RLT with ^177^Lu-PSMA-I&T were mild in East Asians, and no treatment was stopped due to side effects, which is comparable with other studies [16,18,22]. Salivary glands are radiation-sensitive organs due to significant PSMA expression (27). Previous and current studies found high physiological uptake in the salivary glands of patients undergoing ^68^Ga-PSMA PET-CT and post-therapy scintigraphy, which could cause xerostomia and impair QOL (28, 29). However, only 15% patients in the current study experienced mild to moderate xerostomia after relatively high-dose treatment. Oral potassium perchlorate and vitamin C reduced uptake by the salivary glands and promoted clearance, and these medications may thus have reduced the incidence of xerostomia. Preclinical study is currently being conducted to confirm this assumption. No other short or long-term side effects (nausea, vomiting, diarrhea, etc.) have been observed in any patients.

In the current study, 70% of patients developed grade 1–4 haematotoxicity, and only 20% worsened to grade 3–4 haematotoxicity. Adverse events were slightly higher than in other mCRPC cohorts (30); however, this may be because some patients had late-stage disease and failed to other treatment strategies. Progressive mCRPC is prone to bone marrow invasion, leading to extensive damage to granulocytes, red blood cells, and platelets. The included patients had already undergone comprehensive treatment and eventually developed mCRPC, and 19 patients had already developed haematological toxicity, including two with grade 3 haematotoxicity prior to PSMA RLT, suggesting that this transient haematological toxicity remains controllable. No significant liver or kidney toxicity was reported in our study.

Tumour burden on whole-body PSMA-TV and TL-PSMA has been reported to be consistent with tumour response (16, 26). In this study, no significant correlation was found between PSMA PET (SUVmax, SUVpeak, T/B, PSMA-TV, and TL-PSMA) and tumour response to PRLT, this phenomenon might be related with the relatively small population in this study. However, ^68^Ga-PSMA PET/CT was valuable for identifying biomedical recurrence and the remaining tumour, which is helpful for the selection of further treatment. In addition, previous treatment had no impact on tumor response, which was consistent with other study (16).

The impact of baseline laboratory results on PSA response was also assessed. We found that increased AST, ALP, and LDH were negative predictors of a PSA response. It was reported that elevated LDH increased the risk of progression under PSMA RLT (31). In malignant tumours, increased LDH reflects the tumour load and tumour progression, making it valuable for risk stratification in prognostic models and for guiding clinical treatment. In this study, univariate logistic regression analysis showed that mCRPC patients with LDH >261 U/L had a relatively poor PSA response during treatment compared with patients with an LDH <261 U/L, while abnormally elevated LDH during PRLT also indicated a relatively poor treatment outcome. Several researchers have reported that high ALP and LDH were associated with poorer survival (32, 33). Moreover, this study also reflected that overweight (BMI>24) is correlated positively with disease progression after RLT. This is the first time that the correlation between BMI and treatment response was observed, and larger studies are needed to validate the finding. Herein, patients with relevant risk factors (overweight and elevated AST, ALP, and LDH) should be closely monitored to allow their therapy to be adjusted in case of disease progression.

This study had some limitations. First, the study was carried out at a single institution in a relatively small population, which might lead to some bias. Second, the primary endpoint for efficacy was based on PSA level and imaging response, but not all patients underwent ^68^Ga-PSMA-11 PET/CT after each cycle. Third, personalized dosages were used, which might affect treatment efficacy. However, this real-world data showed that most patients benefited from ^177^Lu-PSMA RLT, with improved QOL. In addition, the recruited patients were still being followed-up at the time of analysis and survival data were not available. Further studies are warranted to clarify the optimal dose and cycle interval to maximize the benefits of PRLT.

## Conclusions

This prospective single-arm clinical trial demonstrated that ^177^Lu-PSMA-I&T RLT achieve favourable response and significantly improved QOL in East Asian patients with mCRPC with well tolerance, which associates with significant PSA decline and tumor remission including adenopathy and bone metastasis. Furthermore, a subgroup analysis validated increased BMI, AST, ALP, and LDH were independent predictors of progression after RLT outcome. ^177^Lu-PSMA-I&T RLT might thus be an alternative treatment for mCRPC.

## Data Availability Statement

The original contributions presented in the study are included in the article/supplementary material. Further inquiries can be directed to the corresponding authors.

## Ethics Statement

This prospective study was approved by the local ethics committee at Nanjing Medical University (KY20171208-03, Nanjing, China). The patients/participants provided their written informed consent to participate in this study. Written informed consent was obtained from the individual(s) for the publication of any potentially identifiable images or data included in this article.

## Author Contributions

All authors listed have made a substantial, direct, and intellectual contribution to the work and approved it for publication.

## Funding

This research was supported by grants from Jiangsu Provincial Key Research and Development Program (BE2021605), National Natural Science Foundation of China (92049111,82003532,11805104 and 81570613), Jiangsu Provincial Frontier Grant (BE2017612), Nanjing Science and Technology Development Project (201911042) and Nanjing Medical Foundation (ZKX17027). All the funding supported equally in the design of the study and collection, analysis, and interpretation of data and in writing the manuscript.

## Conflict of Interest

The authors declare that the research was conducted in the absence of any commercial or financial relationships that could be construed as a potential conflict of interest.

## Publisher’s Note

All claims expressed in this article are solely those of the authors and do not necessarily represent those of their affiliated organizations, or those of the publisher, the editors and the reviewers. Any product that may be evaluated in this article, or claim that may be made by its manufacturer, is not guaranteed or endorsed by the publisher.
